# Paradoxical Arthralgia Secondary to Anti-Tumor-Necrosis-Factor Alpha Therapy in Crohn’s Disease

**DOI:** 10.7759/cureus.15835

**Published:** 2021-06-22

**Authors:** Aswani Thurlapati, Siva Santosh Kumar Gandu, Shreedhar Kulkarni

**Affiliations:** 1 Internal Medicine, Louisiana State University Health Shreveport, Shreveport, USA

**Keywords:** infliximab, arthralgia, crohn's disease, anti-tnf therapy

## Abstract

The current treatment of choice for polyarthralgia in Crohn’s disease consists of disease-modifying agents and anti-inflammatory therapy, such as anti-tumor-necrosis-factor alpha inhibitors like infliximab. However, here we report the case of a patient with longstanding Crohn’s disease, who developed polyarthritis after receiving only one dose of infliximab.

A 57-year-old male with a past medical history of Crohn's disease and stage 1 colon cancer was admitted to our hospital with complaints of polyarticular polyarthralgia, stiffness, and restriction of movements at the joints that started one day prior to admission. It initially began in bilateral wrists, impairing him to hold objects, then spread to bilateral ankles, causing him to fall, and finally affected his jaw, leading to inability to chew or articulate. He received the first dose of infliximab infusion 10 days prior to admission. Labs revealed elevated anti-infliximab antibody levels with low infliximab drug levels. He was treated with steroids, azathioprine, and non-steroidal anti-inflammatory drugs with discontinuation of infliximab. On follow-up, he was initiated on vedolizumab for maintenance of Crohn’s disease and did not develop similar complaints again.

Our patient had neither had pre-medication antibodies and positive anti-nuclear antibody, nor received the medication for a long duration as proposed in various studies. He developed severe symptoms affecting the majority of axial skeleton from face to feet just after receiving one dose of infliximab. This suggests that further studies in regard to pathophysiological mechanisms and the dose and duration in correlation to symptoms need to be performed for a better understanding of this disease entity.

## Introduction

Spondyloarthropathy involving the axial skeleton is the most common form of extraintestinal manifestation of Crohn's disease and is seen in approximately 30% of the patients [1]. The current treatment of choice consists of disease-modifying agents and anti-inflammatory therapy, such as anti-tumor-necrosis-factor alpha (anti-TNF) inhibitors like infliximab. However, here we report the case of a patient with longstanding Crohn’s disease, who developed polyarthritis after receiving infliximab. 

## Case presentation

A 57-year-old male with a past medical history of Crohn's disease and stage 1 colon cancer presented to the ED with chief complaints of joint pain and stiffness for one day prior to admission. His symptoms began in the bilateral wrists and later spread to his ankles, knees, and jaw. He described a sharp, 10/10, constant and unremitting pain, which aggravated on movement and relieved mildly with ibuprofen at all the mentioned joints. He also complained of significant stiffness leading to restriction of movements. He was unable to hold objects in his hands, chew food, or articulate speech, impairing him to eat or speak. He later developed difficulty ambulating, leading to mechanical falls. He denied trauma, fever, chills, rash, night sweats, weight loss, abdominal pain, diarrhea, constipation, hematochezia, melena, dysuria, urethral discharge, recent history of travel or sick contacts, and reported no similar joint pains in the past. He also denied similar complaints in his family and reported no known drug allergies. He is compliant with long-term budesonide 9 mg, but due to worsening abdominal pain and diarrhea, he has been recently initiated on infliximab therapy for maintenance of Crohn’s disease. He received his first dose of infliximab infusion 10 days before admission. On physical examination, bilateral wrists and ankles were noted to be in flexion deformity with tight clenching of the jaw. Tenderness, increased localized warmth, and decreased active and passive range of movements of the distal interphalangeal joints, proximal interphalangeal joints, shoulder, and bilateral knee joints were noted. On speaking, a muffled speech was noted due to severe jaw stiffness and inability to open the mouth. In the ED, the patient received IV morphine and hydrocodone-acetaminophen, which provided no relief. Lab values were significant for white blood cell count 13.59k/µL, C-reactive protein 3.69 mg/dL, erythrocyte sedimentation rate 23 mm/h, anti-infliximab antibody (normal <50 U/mL) 416 U/mL with a pre-medicated value of <0.22 U/mL, infliximab drug level (normal >5 mcg/mL) 2.7 mcg/mL with a pre-medicated value of 0.4 mcg/mL, negative anti-nuclear antibody (ANA), and negative anti-double-stranded DNA antibody (anti-dsDNA). Imaging of the joints was unremarkable. He had significant improvement with IV ketorolac, IV methylprednisolone 40 mg, and azathioprine 150 mg, allowing him to return to baseline motor function. On follow-up with gastroenterology, he was discontinued on infliximab and initiated on vedolizumab for maintenance of Crohn’s disease with no recurrent polyarthritis. 

## Discussion

Polyarthralgia could be caused by myriad conditions ranging from self-limiting viral prodrome to disabling rheumatological conditions. One disease known to be associated with polyarthritis, enthesitis, and dactylitis under the spectrum of seronegative spondyloarthropathy is Crohn’s disease. Since the early 2000s, studies suggest that the mainstay of treatment shown to improve arthritis in Crohn’s disease is anti-TNF inhibitors, such as infliximab [2,3]. However, our patient had developed polyarthritis after the initiation of infliximab, even though he reported significant improvement in his abdominal pain and diarrhea. Infliximab is the first anti-TNF agent that has been introduced in the 1990s for rheumatoid arthritis, psoriatic arthritis, and later inflammatory bowel disease. It has a favorable safety profile, barring contraindication in certain conditions. Unexpectedly, few case reports showed infliximab to cause dermatitis and arthritis, bringing into notion paradoxical adverse effects. Skin manifestations are the most common paradoxical events ranging from 8% to 22% [4-6]. However, polyarthralgia may be seen in 1-2%. Symptoms typically occur five to seven days after infusion but can range up to 12 days. 

The key pathophysiology proposed in the development of these manifestations consists of immune dysregulation, cell-lysis-induced inflammatory storm, T-cell-induced B-cell antibody formation, underlying increased susceptibility to autoimmune phenomena, and type 3 delayed hypersensitivity reaction [7]. It is more common in patients with high ANA and anti-dsDNA antibodies, suggesting that patients with more susceptibility to autoimmune phenomena tend to develop paradoxical manifestations [7]. A study performed at Johns Hopkins Arthritis Center revealed that an antibody concentration >8.0 g/mL before infliximab infusion predicted a shorter duration of response and a higher risk of infusion reactions with a relative risk of 2.4. Also, infliximab concentrations were significantly lower with a median of 1.2 in patients with infusion reactions [8]. Studies also determined greater antibody formation in patients on monotherapy and likely not receiving concomitant immunosuppressive therapy [9,10]. However, no studies were noted to indicate the correlation between the duration and the dose of treatment on the development of the antibodies and paradoxical adverse effects. 

Effective therapeutic management of paradoxical arthralgia has not been established. Studies suggest that neither change in the dose or frequency of administration, nor switch to different anti-TNF agents showed any effect. However, initiation of steroids and restarting immunomodulators were efficacious in few patients [11,12]. Studies also suggest that switching to ustekinumab or vedolizumab was more effective than switching to another anti-TNF agent [1]. Hence, in our patient, we decided to continue azathioprine and initiate vedolizumab, an anti-integrin monoclonal antibody. Vedolizumab is noted to have lower immunogenicity, and as seen in the VARSITY trial it is superior to adalimumab in patients with ulcerative colitis [13,14]. Recently in December 2020, a retrospective study performed at MD Anderson comparing vedolizumab versus infliximab in Crohn’s disease suggested vedolizumab to have equal efficacy, lower colitis recurrence, less cancer progression, with better overall survival at 40 months [15]. 

## Conclusions

Our case suggests that patients can have infliximab-related post-treatment arthralgia even in the absence of pre-medication antibodies, ANA, anti-dsDNA, and long duration of treatment, which are all proposed mechanisms. This suggests that further studies regarding pathophysiological mechanisms and the dose and duration correlation to symptoms need to be performed for a better understanding of this disease entity. These adverse effects should be kept in mind before initiation of anti-TNF therapy. 

## References

[1] Alivernini S, Pugliese D, Tolusso B (2018). Paradoxical arthritis occurring during anti-TNF in patients with inflammatory bowel disease: histological and immunological features of a complex synovitis. RMD Open.

[2] Herfarth H, Obermeier F, Andus T, Rogler G, Nikolaus S, Kuehbacher T, Schreiber S (2002). Improvement of arthritis and arthralgia after treatment with infliximab (Remicade) in a German perspective, open-label, multicenter trial in refractory Crohn’s disease. Am J Gastroenterol.

[3] Kaufman I, Caspi D, Yeshurun D, Dotan I, Yaron M, Elkayam O (2005). The effect of infliximab on extraintestinal manifestations of Crohn's disease. Rheumatol Int.

[4] Cullen G, Kroshinsky D, Cheifetz A, Korzenik JR (2011). Psoriasis associated with anti-tumour necrosis factor therapy in inflammatory bowel disease: a new series and a review of 120 cases from the literature. Aliment Pharmacol Ther.

[5] Rahier JF, Buche S, Peyrin-Biroulet L (2010). Severe skin lesions cause patients with inflammatory bowel disease to discontinue anti-tumor necrosis factor therapy. Clin Gastroenterol Hepatol.

[6] Cleynen I, Van Moerkercke W, Juergens M, Ballet V, Drobne D, Vandecandelaere P (2010). Anti-TNF induced cutaneous lesions in IBD patients: characterization and search for predisposing factors. Gut.

[7] Vermeire S, Noman M, Van Assche G (2003). Autoimmunity associated with anti-tumor necrosis factor alpha treatment in Crohn's disease: a prospective cohort study. Gastroenterology.

[8] Center Center, A. (2012, August 29 (2021). Johns Hopkins Arthritis Center. In Crohns Disease, the Development of Antibodies to Infliximab (Remicade) Is Associated with Shorter Duration of Clinical Response and More Infusion Reactions. https://www.hopkinsarthritis.org/arthritis-news/in-crohns-disease-the-development-of-antibodies-to-infliximab-remicade-is-associated-with-shorter-duration-of-clinical-response-and-more-infusion-reactions/.

[9] Colombel JF, Sandborn WJ, Reinisch W (2010). Infliximab, azathioprine, or combination therapy for Crohn's disease. N Engl J Med.

[10] (2012). Measurement of infliximab and anti-infliximab antibody levels can help distinguish maintenance versus loss of response. Gastroenterol Hepatol (N Y).

[11] Sharon D-B (2020). Effective management of autoimmune arthralgia arising from anti-TNF. Inflamm Bowel Dis.

[12] Ally MR, Betteridge JD, Veerappan GR (2012). Delayed hypersensitivity reaction with infliximab: unusual to occur after initial dose. Inflamm Bowel Dis.

[13] Roda G, Chien Ng S, Kotze PG (2020). Crohn's disease. Nat Rev Dis Primers.

[14] Sands BE, Peyrin-Biroulet L, Loftus EV Jr (2019). Vedolizumab versus adalimumab for moderate-to-severe ulcerative colitis. N Engl J Med.

[15] (2020). Managing Checkpoint Inhibitor-Mediated Colitis: Vedolizumab vs Infliximab. https://ascopost.com/issues/december-10-2020/managing-checkpoint-inhibitor-mediatedcolitis/.

